# Use of extracorporeal membrane oxygenation for pediatric post-traumatic pulmonary hemorrhage: A Case report and literature review

**DOI:** 10.1016/j.tcr.2025.101255

**Published:** 2025-10-02

**Authors:** Abdulrahman Alwahbi, Abdulrahman Alhowaiti, Abdullah Akkam, Mohammed Tairi, Bandar Alanazi, Saleh Alshehri, Robeyh Assiri

**Affiliations:** a Department of Pediatric Emergency, King Saud Medical City, Riyadh, Saudi Arabia; b Department of Pediatric Intensive Care Unit, King Saud Medical city, Riyadh, Saudi Arabia; c Department of pediatric Emergency, King Abdullah Specialized Children Hospital, Riyadh, Saudi Arabia

**Keywords:** Pulmonary Hemorrhage, Chest Trauma, Lung Contusion, Extracorporeal Membrane Oxygenation, Pediatrics, Emergency

## Abstract

Chest trauma in children presents unique diagnostic challenges due to physiological and anatomical differences from adults. While pulmonary contusion remains the most common injury, meticulous evaluation with CT scan is crucial to rule out occult pathologies and ensure timely intervention for potential complications like alveolar hemorrhage.

Pediatric chest trauma mandates a tailored management approach considering their delicate physiology. Early initiation of high-flow oxygen, judicious ventilatory support for acute respiratory distress, and proactive fluid management are essential, while pain control and hemodynamic monitoring remain critical throughout the recovery process.

Here, we report a challenging case of a 6-year-old male child presenting to the Pediatric Emergency Department with acute moderate-to-severe respiratory distress that was successfully treated with extracorporeal membrane oxygenation.

The child was brought to our emergency department with only history of mild head trauma that occurred 2 h before presenting to the hospital. After triaging as Canadian Triage and Acuity Scale (CTAS) II, the child was managed in line with acute respiratory distress via ATLS abroach. We ruled out head, cervical spine, and other evidence of invasive chest as well as gross abdominal injuries, by ATLS abroach and adjuncts such as point-of-care ultrasound and chest and abdomen X-rays and PAN CT. Although the initial venous blood gas analyses were suggestive of mixed respiratory an metabolic acidosis, the CXR and the chest CT revealed that the child had significant lung parenchymal injury in the form of bilateral fluffy pulmonary infiltrates. This case indicates that even an uncertain history and absence of physical finding, chest blunt trauma causing lung injury, leading to severe manifestations and sometimes fatal complications such as pulmonary contusion, hemorrhage, and ARDS.

## Introduction

In developed countries, trauma is the primary cause of death for children between the ages of one and fourteen, with thoracic trauma being the second most common fatal injury after head trauma. While children experience thoracic injuries less frequently than adults, these injuries remain a significant source of morbidity and mortality. Diverse issues such as rib fractures, lung damage, hemothorax, pneumothorax, mediastinal injuries, and others may present individually or in combination. Direct lung injury frequently manifests as non-anatomical consolidation zones, often without accompanying rib fractures, chest wall contusions, or other externally observable anatomical correlates of lung trauma [1] [2].

Approximately 5 % to 12 % of pediatric trauma admissions involve thoracic trauma. Despite this relatively low percentage, these injuries are disproportionately associated with high morbidity and mortality compared to other types of injuries [3]. The infrequency of these injuries contributes to the complexities of managing them in both emergency department and intensive care unit settings.

The physiologic consequences, such as the dreaded complication of alveolar hemorrhage and pulmonary parenchymal destruction, usually manifest within a few hours of the trauma and can take up to seven days to recover [4].

VenoVenous-ECMO is a valuable tool for managing severe respiratory failure with preserved cardiac function. It provides lung-protective ventilation and gas exchange, facilitating rest and recovery of the native lungs [5].

## Case report

**Fig. 1:**
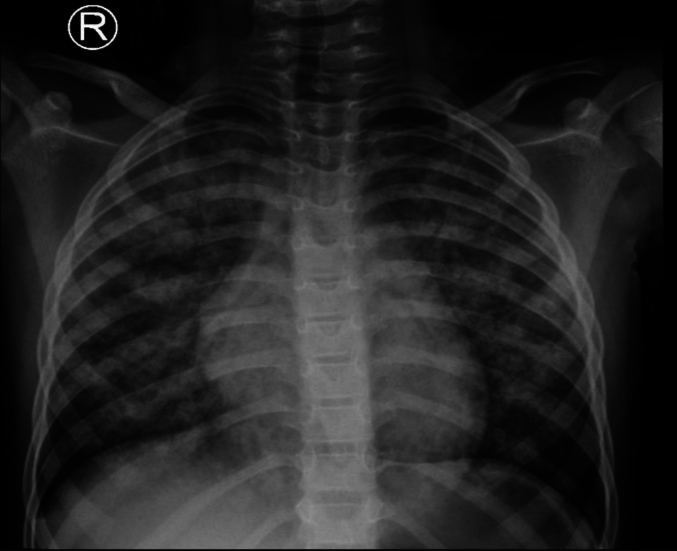
Diffuse bilateral pulmonary opacities with patchy and confluent infiltrates, suggestive of pulmonary contusions.

6 years old Malian boy with no significant medical history. Presented accompanied by his family after he fell down from his bed into wall on his occipital area, after that he developed LOC for few seconds then became drowsy and not able to move. Upon physical examination the young boy had a poor general appearance, more on the respiratory examination he was dyspneic and tachypneic breathing, pulse of 175 beats/min, and intercostal and subcostal retractions with paradoxical chest and abdominal breathing, auscultation showed a diffuse, crepitant rales in all areas of bilateral lungs. A chest x-ray showed a diffuse, bilateral opacities in all of lung fields. Fig. 1.

Initial laboratory analysis where with normal ranges for age. Venous blood gases: pH 7.00, HCO 17.30 mmol/L, PaCO2 70 mmHg, and PaO2 42 mmHg B.E − 14.9. Trauma *E*-FAST was conducted and was unremarkable.

**Fig. 2:**
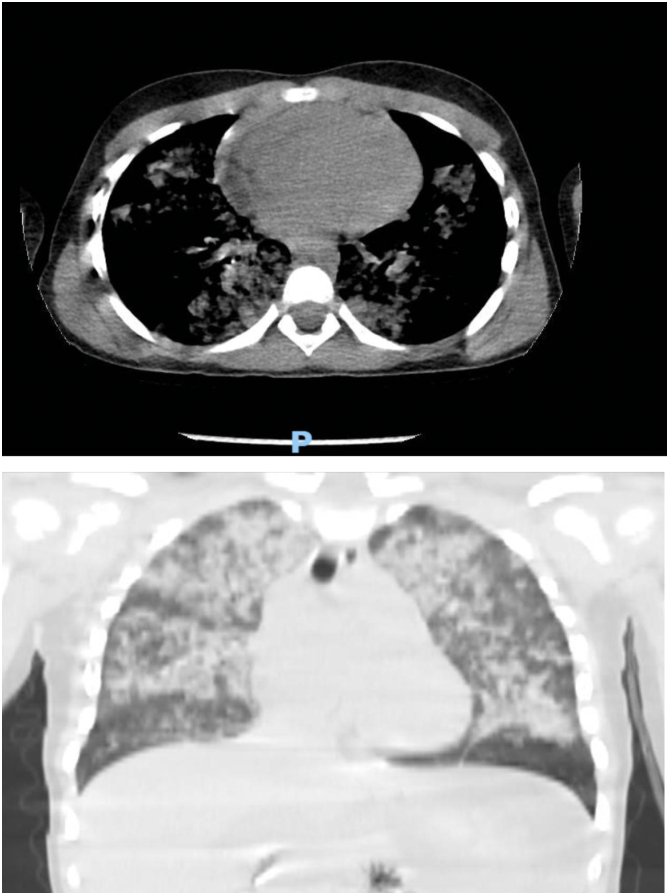
Near total consolidation with air bronchograms seen in both lungs.

With absence of clear history and presence of this clinical picture, the child was started on bilevel positive airway pressure. And a fluid resuscitation was established through IV line. After the vitals were more stabilized, the child was shifted to computerized tomography scans as pan scan was established. The head, cervical spine abdomen and pelvis scans were unremarkable. Chest scan revealed Diffuse widespread lung parenchymal nodular ground glass opacities in both sides. Fig. 2.

After a couple hours he child's respiratory condition worsened and he developed bleeding from mouth and nostril, suspension for pulmonary hemorrhage and acute lung injury was raised, boy was then intubated and admitted to ICU. Repeated imaging showed early signs of ARDS and even though child was connected high frequency oscillatory ventilation there was no improvement and child's condition worsened and repeated investigations showed drop in hemoglobin from 12 to 5 g/dL.

**Fig. 3:**
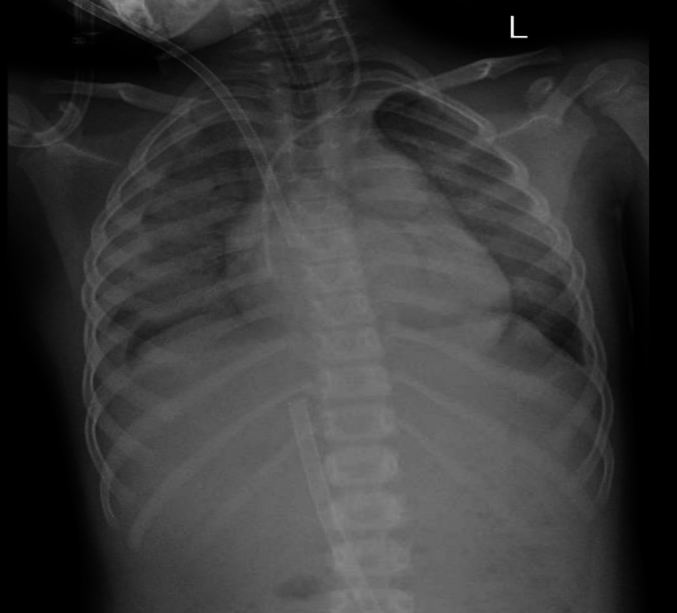
Acute respiratory distress syndrome and ECMO was established.

**Fig. 4:**
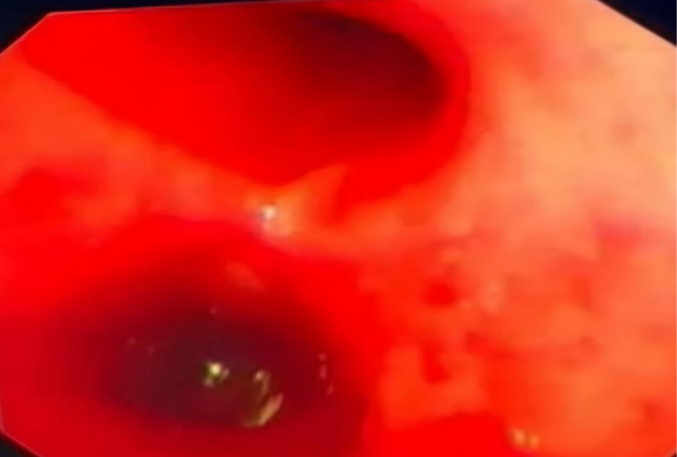
Bronchoscopy showing alveolar hemorrhage.

Extracorporeal membrane oxygenation team contacted and child was started on ECMO, fig. 3 then child condition stabilized and diagnostic bronchoscopy was done that showed massive pulmonary hemorrhage. Fig. 4.

Over next 48 h after starting ECMO child condition stabilized furthermore.

**Fig. 5:**
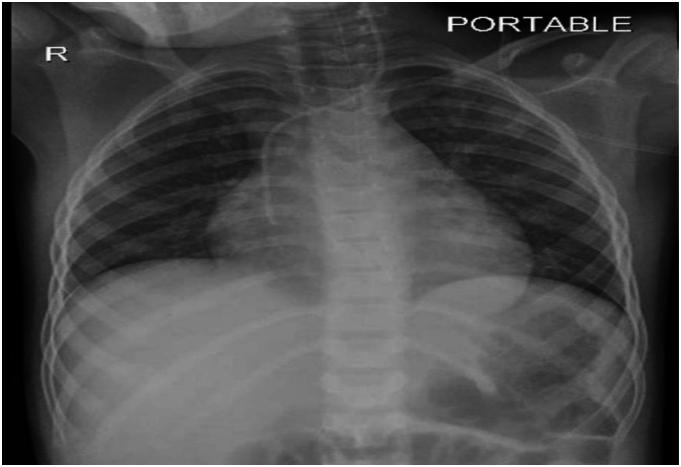
Chest x-ray showing dramatic improvement of lungs parenchyma.

Child was kept on ECMO for nearly a week then stopped and child was extubated after 10 day from intubation then shifted to general ward to complete management. (fig. 5).

## Discussion

As discussed in our case, the child presented with mixed acidosis, hypoxemia and an abnormal pattern of breathing, with evidence of ALI or a mild or evolving form of ARDS, which subsequently required ECMO. Even though there was no clear history of trauma and no obvious physical suggestion of injury, this case had developed bilateral lung contusions with evident radiological and clinical manifestations of significant ALI, the presentation was remarkable and quite atypical. Pulmonary contusion is the most common chest injury in children, occurring in more than half of all blunt chest traumas [6], and many victims do not have evidence of external chest wall trauma [7]. We noted similar findings in our unique observation in the index case, which presented with unclear history and no noticeable external injuries.

Hemorrhage into the pulmonary parenchyma leads to contusion pathophysiology that worsens for 24–48 h and then generally resolves seven days after the injury [8]. The impact of injury should be severe enough to cause disruption of the capillaries of the alveolar walls and septa, causing leakage of blood into the alveolar spaces and interstitial lung tissue [9], which in our case was very unclear and difficult to explain with such no clear history and no information on the mechanism of injury. However, Chest X-rays might overlook early signs, especially soon after the injury. Studies show only 47 % of chest X-rays are accurate upon admission, compared to 92 % after 24 h. In contrast, chest CT scans are the gold standard for diagnosis, offering near-perfect accuracy (100 % sensitivity) but with some limitations in pinpointing exactly where the injury is (40 % specificity). Overall, chest CT provides the clearest picture for diagnosing lung contusions caused by blunt chest trauma [8].

The treatment of pulmonary contusions remains majorly supportive with good oxygenation and adequate analgesia support. There is usually a very minimal need for invasive and mechanical ventilation [8]. However in sever cases and in patient with sever ALI and ARDS similar to our case ECMO was successfully used and showed great promising outcomes.

## Conclusion

Acute respiratory distress syndrome has a high mortality among trauma patient. For challenging cases with limited history and no obvious physical signs, maintaining a high level of suspicion and initiating early intervention can significantly enhance the chances of a positive prognosis.

While not yet widely adopted, Recent studies have shown promising results for using ECMO as a lifeline for trauma patients with severe lung damage (ARDS) arising from pulmonary contusion., this approach offers the potential for improved survival rates in critically ill population.

## CRediT authorship contribution statement

**Abdulrahman Alwahbi:** Resources, Formal analysis, Writing – original draft, Conceptualization, Data curation, Methodology. **Abdulrahman Alhowaiti:** Resources, Formal analysis, Writing – original draft, Conceptualization, Data curation, Methodology.

## Declaration of competing interest

The authors declare that they have no known competing financial interests or personal relationships that could have appeared to influence the work reported in this paper.
